# Differential Soft Sensor-Based Measurement of Interactive Force and Assistive Torque for a Robotic Hip Exoskeleton

**DOI:** 10.3390/s21196545

**Published:** 2021-09-30

**Authors:** Sun’an Wang, Binquan Zhang, Zhenyuan Yu, Yu’ang Yan

**Affiliations:** School of Mechanical Engineering, Xi’an Jiaotong University, No. 28, Xianning West Road, Xi’an 710049, China; bqzhang@stu.xjtu.edu.cn (B.Z.); yzy19961114@stu.xjtu.edu.cn (Z.Y.); yanyuang@stu.xjtu.edu.cn (Y.Y.)

**Keywords:** wearable robots, wearable sensor, soft force sensor, hip exoskeleton

## Abstract

With the emerging of wearable robots, the safety and effectiveness of human-robot physical interaction have attracted extensive attention. Recent studies suggest that online measurement of the interaction force between the robot and the human body is essential to the aspects above in wearable exoskeletons. However, a large proportion of existing wearable exoskeletons monitor and sense the delivered force and torque through an indirect-measure method, in which the torque is estimated by the motor current. Direct force/torque measuring through low-cost and compact wearable sensors remains an open problem. This paper presents a compact soft sensor system for wearable gait assistance exoskeletons. The contact force is converted into a voltage signal by measuring the air pressure within a soft pneumatic chamber. The developed soft force sensor system was implemented on a robotic hip exoskeleton, and the real-time interaction force between the human thigh and the exoskeleton was measured through two differential soft chambers. The delivered torque of the hip exoskeleton was calculated based on a characterization model. Experimental results suggested that the sensor system achieved direct force measurement with an error of 10.3 ± 6.58%, and torque monitoring for a hip exoskeleton which provided an understanding for the importance of direct force/torque measurement for assistive performance. Compared with traditional rigid force sensors, the proposed system has several merits, as it is compact, low-cost, and has good adaptability to the human body due to the soft structure.

## 1. Introduction

Great progress has been made in wearable lower-limb exoskeletons over the past two decades [1,2,3]. These emerging robots are getting extensive attention nowadays due to the significant potentials in many fields. According to the literature [4,5], wearable lower-limb exoskeletons can be divided into three main categories based on application scenarios: weight-bearing in military and industrial settings, rehabilitation for people with pathological gaits, and gait assistance for the elderly or even healthy people. Many countries are facing an ageing population, and there are lots of recent studies which help the elderly improve their quality of life by developing compact and portable wearable exoskeleton robots [1,3,6]. These so-called partial-assist exoskeletons are expected to be used for rehabilitation and daily gait assistance [2,7,8,9,10]. Furthermore, several studies suggest encouraging results in reducing walking metabolic costs and improving gait performance [3,11], which has great potential in applications such as health care, rehabilitation, and gait assistance.

For partial-assist exoskeletons, users generally have partial or full ability to control their lower-limb motions [6]. And the exoskeleton provides a certain proportion of biological torque to reduce the wearer’s metabolic cost and help the wearer walk further. In general, gait assistance exoskeletons need to generate force/torque according to the user’s lower-limb motion. Due to the multiple daily locomotion activities (such as walking, stair ascending/descending and crossing over obstacles), wearable sensors are introduced to detect the gait motion states [12,13,14]. In addition to the complex gait locomotion modes, individual differences also bring significant challenges to partial-assist exoskeletons for keeping synchronization with human movements. Furthermore, current exoskeleton control technologies still can not fully ensure the safety and stability in real-world daily scenes [1,15,16,17]. Since these powered mechatronic devices are in the same physical space with people, human safety and assistance performance of these robots are the two most critical aspects. To this end, it is necessary to monitor the physical interaction force between the exoskeleton and the human body for evaluation of the robot performance and safety control in urgent situations.

Most existing partial-assist exoskeletons are motor-driven [1,3]. A common method of monitoring the physical interaction is based on the delivered torque, which is estimated from the motor current based on parameters and modelling of the motor and its mechanical transmission system. However, the interaction force between robot and human can not be obtained directly by this method, which may make the wearer’s safety unable to be fully guaranteed in some unexpected situations. Besides, the lubrication and wearing position of the mechanical system may also affect the parameters of the calculation model, resulting in errors between the estimated torque and the actual value [18]. In order to address the problems above, several studies introduced wearable sensors to measure human-robot interaction force directly. For example, the exoskeletons in [19,20] adopted torque sensors to measure the assistive torque directly. However, this method may increase the hardware cost of the robot because dynamic torque sensors are usually expensive. The interaction force still cannot be measured directly since the torque sensor is usually mounted on the output shaft of the drive unit. In order to directly obtain the interaction force in wearable robots, Choi et al. [18] proposed a compact force sensor for a gait assistance exoskeleton based on force-sensitive resistors (FSR). The sensor system can directly measure the interaction force between the human thigh and hip exoskeleton. Beil et al. [21] introduced three-dimensional force sensors to measure the interaction force of a knee exoskeleton. However, the contact part between the measurement system above and the human body is rigid. Thus, the measurement results may be affected by the contact area between the rigid element and the human body, for the reason that it is usually difficult to ensure good contact between the rigid sensor surface and irregular surface of the human body (clothes).

Another alternative approach is to develop soft wearable sensors. For example, Cheng et al. [22] proposed a soft fabric-based pneumatic sensor to detect bending angles and contact force in human-robot interaction. The developed sensor system is aimed at addressing the sensing problem of soft actuators. The maximum force of the designed force sensor is 35 N. Besides, Choi et al. [23] proposed a novel three-axis soft force sensor, with a maximum force range of 13 N along the vertical axis. Araromi et al. [24] proposed a hybrid carbon fiber-textile compact force sensor, which was developed for load measuring of exosuits. This sensor was able to measure the loaded tension of the exosuit with the range of 300 N. Compared with rigid sensors, soft sensors can better adapt to the human body and make people feel comfortable. Despite the great prospects of soft sensors in human-robot interaction, wearable soft force sensors for partial-assist exoskeletons remain an open problem.

The scope of this paper is to develop a soft pneumatic force sensor system, which is mainly used to measure interaction force detection between the exoskeleton and human thigh. As a first attempt, we conducted the trial of controlling a hip exoskeleton using a preliminary soft sensor system in our previous work [25]. However, it was found that the developed sensor system needs to spend much time on the exoskeleton for adjustment in previous experiments. Therefore, in this paper, we redesign the mechanical structure of the exoskeleton thigh frame. Furthermore, air chambers of different shapes and materials were fabricated and tested, guiding the design of wearable soft sensors on the exoskeleton.

The contributions of this work include the following aspects.

- A wearable soft sensor system for direct force measurement of a hip exoskeleton is designed, with merits of low-cost, compact, and well adapted to the human body.
- The characteristics of air chambers with different materials and shapes were compared and tested, providing a guidance for the design of wearable soft sensors.
- The developed soft sensor was integrated on a hip exoskeleton, and evaluation experiments were performed with eight healthy subjects.

The remainder of this paper is organized as follows. In Section 2, the design and fabrication of the soft sensor system are reported. Next, the results and statistical analysis of the validation experiments are presented in Section 3. Subsequently, the performance and limitations of the proposed method are discussed in Section 4. Finally, conclusions and future work directions are given in Section 5.

## 2. Methods and Materials

**Figure 1:**
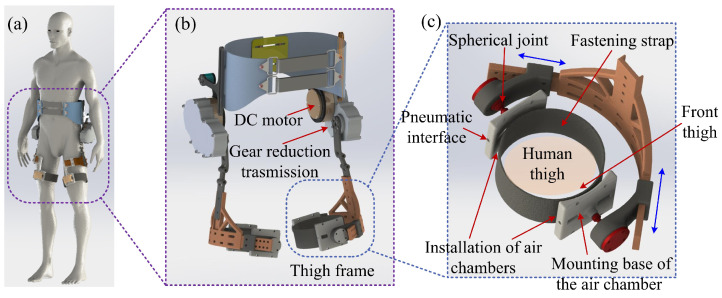
The 3D model of the hip joint exoskeleton and the soft sensor system. (**a**) The composition of the exoskeleton of the hip joint and the design of the wearing structure around the thigh. (**b**) The hip exoskeleton with soft sensors. (**c**) The designed adjustable thigh frame and installation of the developed soft sensor system.

Since the hip exoskeleton is closely worn on the outside of the human body, the installation position is highly related to the measurement purpose of the wearable sensor. In general, the assistance of the exoskeleton is carried out in the sagittal plane of the human body. The interaction between the hip exoskeleton and the human leg is mainly concentrated in the front and back area of the thigh. As a result, the developed soft sensors are installed between the thigh and the exoskeleton. The system design of the hip exoskeleton and sensor installation is presented in Figure 1. The bidirectional (leg lifting and falling) interaction force during locomotion can be measured through two air chambers (shown in Figure 1c). The thigh frame of the hip exoskeleton and the mounting bases of the air chamber are connected through spherical joints, which ensures that the assistive force of the exoskeleton can be better perpendicular to the thigh.

### 2.1. Design and Fabrication of the Soft Force Sensor System

**Figure 2:**
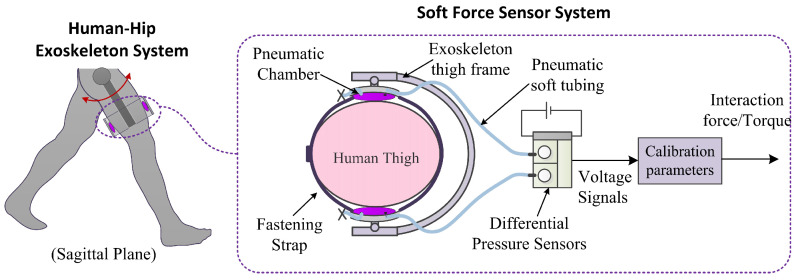
Overall schematic diagram of the developed soft force sensor system.

The overall schematic diagram of the soft sensor system is shown in Figure 2. The dynamic interaction forces are converted into the air pressure changes within a soft pneumatic chamber. Then the air pressure is converted into a voltage signal through a differential air pressure sensor. When the air chamber is subjected to an external force, air within the chamber is compressed. Thus the air pressure rises. These changes increase the output voltage of the air pressure sensor. On the contrary, when the external force acting on the surface of the chamber decreases, the internal air pressure decreases, and the voltage output of the pressure decreases. Subsequently, the voltage signal is filtered, amplitude adjusted and sampled by a developed electronic system. The interaction force information is obtained by the measured voltage signal and a calibration model, and the delivered torque is calculated based on the measured force.

**Figure 3:**
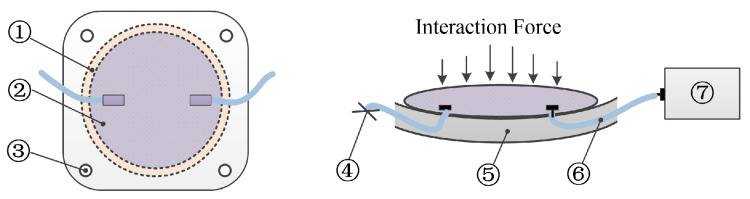
Design of the soft chamber (**left**) and the detailed diagram of its installation (**right**). (1) Thermoplastic pressurized area; (2) Air chamber; (3) Mounting hole of the chamber; (4) Inflation port value; (5) Chamber mounting base; (6) Pneumatic silicone rubber tube; (7) Air pressure sensor.

The design of the soft air chamber is shown in Figure 3. The air chamber is fabricated with fabric thermoplastic polyurethane (TPU) film materials. Two pieces of PTU materials are bonded through a hot pressing process, and the middle part is sealed to form the air chamber. During initialization, the air chamber is inflated with air through the inflation valve, and then the inflation valve is closed. The air chamber is neither inflated nor deflated during force measurement. The initial inflation pressure setting is determined by the characteristics of the air chamber. The chamber, pneumatic tubes, and air pressure sensors are integrated with the exoskeleton thigh frame. Materials and geometric parameters are the key factors affecting the soft sensor characteristics. As a result, we tested four air chambers of different thicknesses and shapes for comparative analysis when fabricating and testing the characteristics of air chambers.

### 2.2. Signal Processing and Electronic System

**Figure 4:**
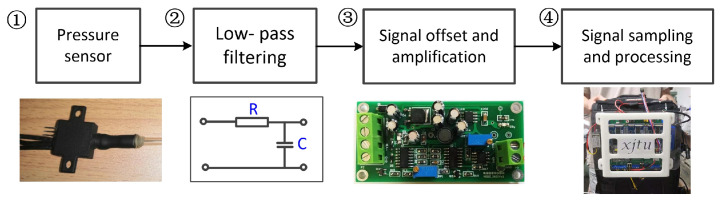
Signal processing and electronic system. (1) The air pressure sensor. (2) RC low-pass filtering circuit. (3) Modular for signal amplitude amplification and offset. (4) Embedded electronic system.

The process of signal sampling and processing for a single air chamber is presented in Figure 4. A differential air pressure sensor (MPX4250, NXP) senses the air pressure within the air chamber and converts the real-time air pressure into a voltage signal. Then this voltage signal passes through a resistance-capacitance (RC) low-pass filtering circuit to remove the high-frequency noise. The cut-off frequency of the RC filter is set close to 100 Hz, which is much higher than the required frequency of human locomotion (below 20 Hz) [26]. Since the voltage signal changes between 0.2 V and 1 V, an analogue circuit for offset and amplification is designed to adjust the signal amplitude range (to the range between 0 V and 3.3 V) for analogue/digital (A/D) signal sampling. After the conversion above, the signal is sampled and processed by a circuit. The electronic system is integrated with the embedded controller of the hip exoskeleton and installed on the wearer’s back through a backpack. Sampling and processing of voltage signals are completed by an ARM-based microprocessor (STM32F407, STMicroelectronics). In the following tests and experiments of the developed soft sensor system, the frequency of A/D sampling is set to 200 Hz.

### 2.3. Pneumatic Calibration and Characterization

**Figure 5:**
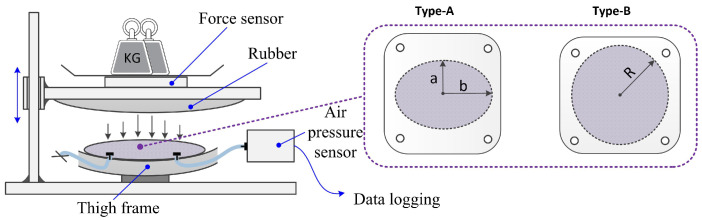
Schematic diagram of soft chamber characteristics test (**left**) and design of different shapes of air chambers (**right**). Geometric parameter: *a* = 23 mm, *b* = 30 mm, *R* = 60 mm.

**Figure 6:**
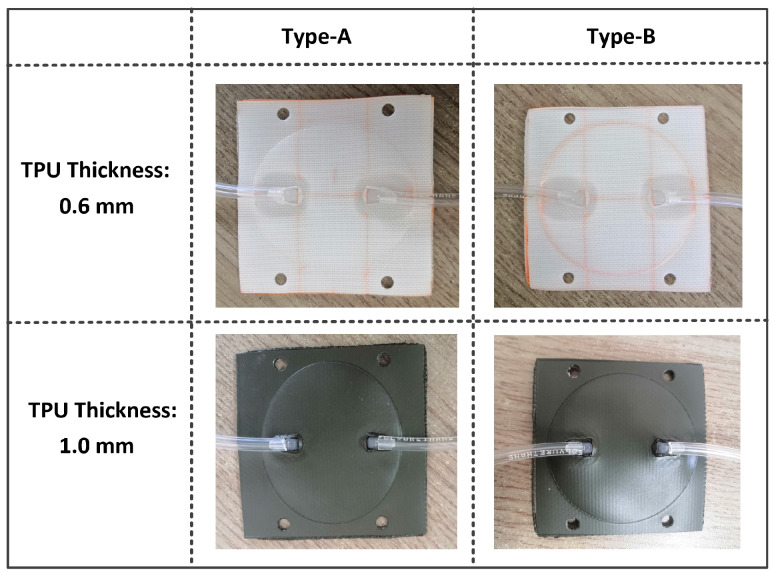
Fabrication of different soft force sensors. “Type-A” and “Type-B” are shown in Figure 5. The two fabric-based TPU materials are different.

In order to obtain the pneumatic characteristics of air pressure within the chamber with external forces, loading force tests were carried out after the fabrication of the soft sensor system. The loading force test process is shown in Figure 5. The static loading force is provided by the gravity of the standard weight. Under different initial inflation pressures and static loading forces, the air pressure values were tested and recorded by applying different standard weights to the air chambers. In order to make the test process close to the actual wearing situation, we fixed the soft chamber on the thigh frame of the exoskeleton and selected rubber materials to contact the upper surface of the air chamber. The use of rubber can emulate the actual situation of human contact with the chamber approximately. In order to obtain the characteristics of different materials and chamber shapes, we designed four different air chambers (shown in Figure 6). In addition, two fabric-based TPU materials, with thicknesses of 0.6 mm and 1.0 mm, respectively, were tested.

**Figure 7:**
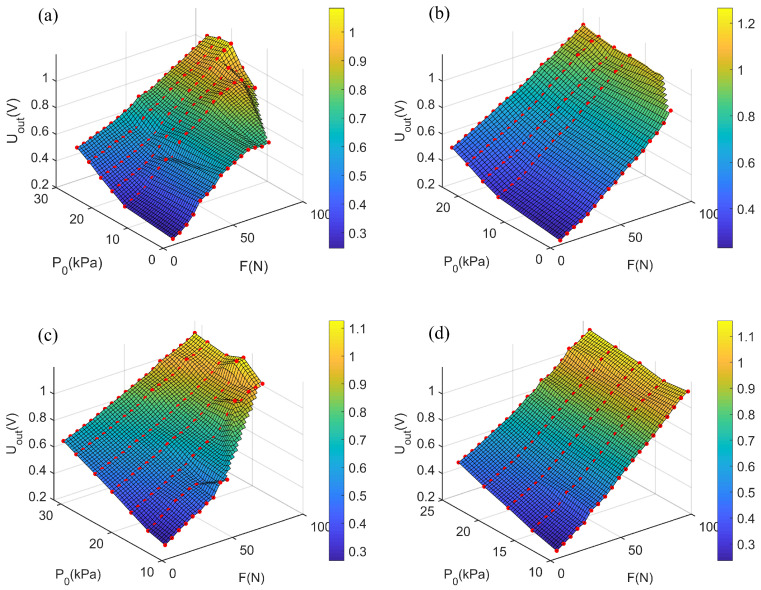
Visualization of the characteristics of different chambers. (**a**) Type-A chamber, with TPU thicknesses of 0.6 mm. (**b**) Type-B chamber, with TPU thicknesses of 0.6 mm. (**c**) Type-A chamber, with TPU thicknesses of 1.0 mm. (**d**) Type-B chamber, with TPU thicknesses of 1.0 mm.

The test results for static loading of different air chambers are visualized in Figure 7. It can be seen that different air chambers have different static characteristics according to the results. In order to determine the most suitable chamber to be used as the soft sensor, several sensing performance aspects were evaluated, including linearity, measurement stability, and measurement range. As shown in Figure 7, Type-B chambers have better linearity in the whole measurement range than Type-A chambers. The measurement range and linearity of Type-A chambers are easily affected by the initial inflation pressure, and the linearity is poor at low inflation pressure. From the perspective of TPU material thickness, the TUP material with a thickness of 0.6 mm has a more extensive measurement range than that with a thickness of 1.0 mm. Besides, the TUP material with a thickness of 0.6 mm can make the chambers consistent under different initial inflation pressures. Based on the analysis above, a Type-B chamber with a TPU thickness of 0.6 mm was selected for the soft force sensing system.

Based on the static characteristic tests above, the characteristic parameters of the selected soft chamber and the electronic system can be determined. The functional relationship between output voltage (offset and amplified), initial inflation pressure and external force can be established, described as:

(1) $$U_{out}=k\left(p_{0}\right)\cdot F+b\left(p_{0}\right)$$

where *k* and *b* are the linear parameters related to the initial pressure $p_{0}$, respectively.

**Figure 8:**
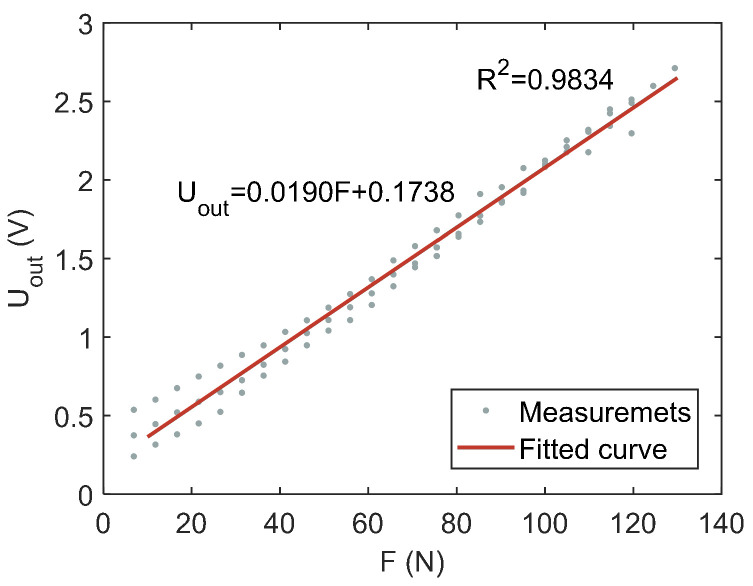
Relationship between force sensor voltage and loading force. Each measurement point represents the average of 200 measurements. $U_{out}$ represents the offset and amplified voltage.

In order to simplify the model, the initial inflation pressure range can be set within the range of 15 kPa to 20 kPa. Thus, the characteristics of the air chamber can be approximately replaced by a linear function (visualized in Figure 8):

(2) $$U_{out}=k_{s}\cdot F+b_{s}$$

where $k_{s}$ and $b_{s}$ are the linear parameters of the simplified model, respectively. $k_{s}$ = 0.019, $b_{s}$ = 0.1738. The fitting correlation coefficient is $R^{2}$ = 0.9834.

**Figure 9:**
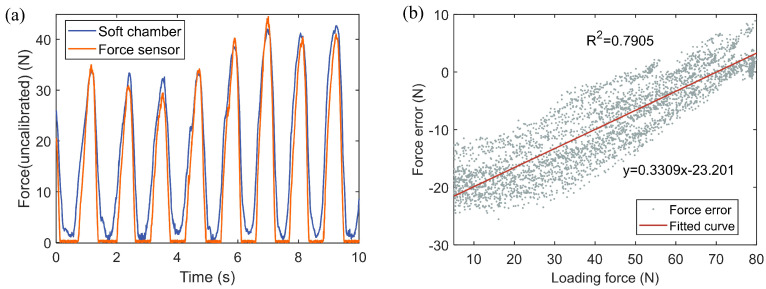
(**a**) Output of the developed force sensor system under dynamic loading force. (**b**) Force errors under different dynamic loading forces (5 N to 80 N).

Based on Equation (2), the loading force on the soft sensor can be measured through the output voltage. And the result is compensated based on the error statistical results in Figure 9b. Thus the measurement result of the force sensor is given by Equation (3).

(3) $$\hat{F}=\frac{U_{out}-b_{s}}{k_{s}}-e_{c}\left(F\right)$$

where $\hat{F}$ is the output of the developed soft sensor. $e_{c}\left(F\right)$ is a error compensation term related to force measurement, which is obtained through a dynamic-loading-force calibration process, which will be introduced in following parts. Due to the manufacturing error of air chambers, the error term $e_{c}\left(F\right)$ of each chamber may be slightly different. However, it can be obtained through the same process above.

**Figure 10:**
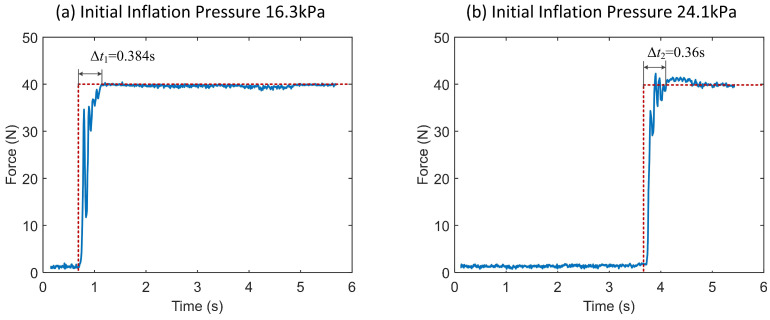
Step loading force test at different initial inflation pressures. The loading forces were given by an electric cylinder loading test bench.

In order to test the performance of the developed sensor under dynamic loading force, a dynamic force with a maximum amplitude of about 80 N was manually loaded on the experimental platform in Figure 5. Part of the temporal curve and measurement performance of the sensor output are shown in Figure 9a (uncalibrated). During dynamic force loading, the outputs of force sensor of reference and the developed soft sensors system were recorded. Through the measured errors, the calibration of the developed sensor was realized. The measurement error and its fitting curve are shown in Figure 9b. And this fitting line is used as the error compensation term $e_{c}\left(F\right)$ of the sensor in (3). Considering the manufacturing error of the air chambers, the above process is repeated for each developed soft sensor to realize parameter calibration. In order to test the time-delay characteristics of the developed sensor system, step force tests were performed under different initial inflation pressures, as shown in Figure 10. The average lag time was within 0.4 s. Based on the experimental tests above, the performance of the developed soft sensor system is summarized in Table 1.

### 2.4. Force and Torque Measuring for a Hip Exoskeleton

**Figure 11:**
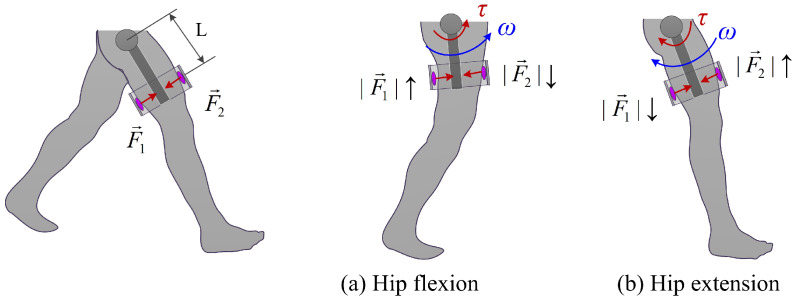
Schematic diagram of human-exoskeleton interaction force/torque measurement. The directions of the interaction forces are defined in the figure.

After completing the chamber test and parameter calibration, the interaction force between exoskeleton and human is measured by the differential force of two chambers in the front and back of thigh, respectively. Before measuring the interaction force and torque, the operation of the exoskeleton was as follows. Firstly, the air chambers were installed on the exoskeleton thigh frame. Then the thigh frame was adjusted to adapt to the wearer’s leg geometry. Due to the use of spherical joints of exoskeleton legs, human legs only exert force with front and rear air chambers. As shown in Figure 11, the interaction force is calculated by:

(4) $$\vec{F}={\vec{F}}_{1}-{\vec{F}}_{2}$$

where ${\vec{F}}_{1}$ and ${\vec{F}}_{2}$ are the measured force by the two soft sensors, respectively. $\vec{F}$ is the resultant force.

The force measurement above was carried out under the assumption that the force of hip exoskeleton was delivered on the human thigh completely. This aspect was guaranteed through the structural design of exoskeleton. Through the spherical joint structure in Figure 1c, the interaction between the exoskeleton and the human thigh is perpendicular to the leg, avoiding the force in other directions.

In addition, the delivered torque can be calculated based on the exoskeleton geometric parameters:

(5) $$\vec{\tau }\left(t\right)=\vec{F}\left(t\right)\cdot L$$

where *L* is length of the exoskeleton leg link (torque arm), *L* = 0.23 m.

## 3. Experiments and Results

**Figure 12:**
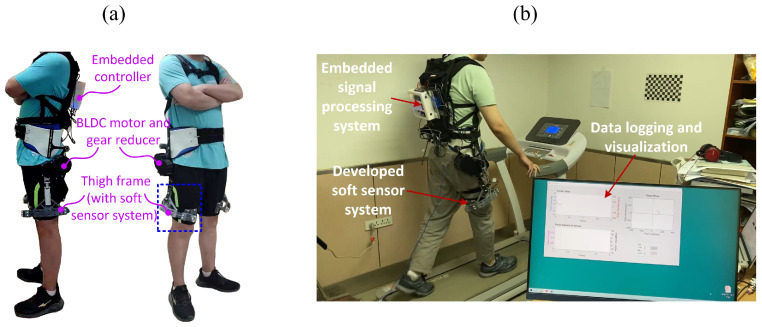
Fabrication of the soft force sensor (**a**), and the characteristic curve of the applied force and output voltage of the force measurement system (**b**).

The developed soft sensor system is integrated into the hip exoskeleton, and the walking test was carried out on the treadmill. The hip exoskeleton is shown in Figure 12a. It was driven by DC motor, and the assistive torque was applied to the hip joint through gear transmission (transmission ratio 12:1). The angle of the thigh was measured by the angle sensor of the hip exoskeleton. And the angular velocity of the thigh was calculated from the temporal derivative of the thigh angle. The assist torque of exoskeleton was generated based on our previous work [27] through a continuous phase variable, which was a monotonically increasing scalar scaled between zero and 2π rad within each stride. The assistive torque is governed by:

(6) $$\tau \left(t\right)=-a_{1}e^{\frac{-{\left(\varphi -\mu_{1}\right)}^{2}}{2{\sigma_{1}}^{2}}}+a_{2}e^{\frac{-{\left(\varphi -\mu_{2}\right)}^{2}}{2{\sigma_{2}}^{2}}}$$

where φ is the phase variable (converted to 0–100%), $\mu_{1}$, $\mu_{2}$ are the phases corresponding to the centre of the torque peak, $\mu_{1}$, $\mu_{2}$, $\sigma_{1}$, and $\sigma_{2}$ are the parameters to refine the assistive toque profile, which were set as different values in the experiments. $a_{1}$ and $a_{2}$ are the amplitudes of Gaussian basis functions, $a_{1}$ = 2.5 Nm and $a_{2}$ = 4 Nm.

In the exoskeleton experiment, a total of 8 healthy subjects participated in the experiment. The subjects information is given in Table 2. Figure 12b presents the settings of the treadmill experiment. The experimental process is as follows. Each experimental subject wore a unilateral hip exoskeleton and walked on the treadmill at different setting speeds (including 3 km/h and 4 km/h, walking 1 min for each speed), and recorded the assistive torque of the exoskeleton and the measure torque of the soft sensor, respectively, in real time. In the experiments, different profiles of the assistive torque were tested. The experiment included a healthy male subject. Informed consent was obtained from the subject involved in the study and written informed consent has been obtained.

**Figure 13:**
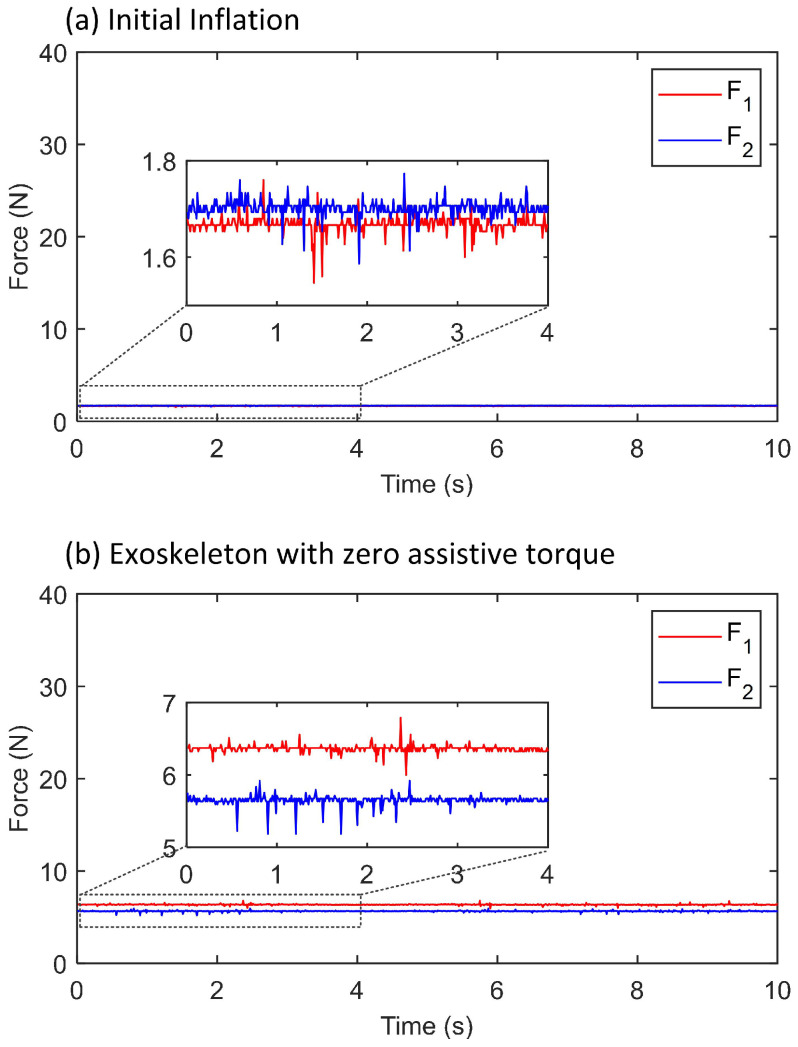
Force measurements during initial stage. (**a**) The soft air chamber were inflated but the exoskeleton was not worn on the subject. (**b**) The exoskeleton was worn on the subject, the thigh frame was fastened by the fastening strap, and the exoskeleton output zero torque.

The outputs of the two soft force sensors after initial inflation and worn on the human body are shown in Figure 13 (for one subject). When the soft air chamber were inflated but the exoskeleton was not worn on the subject, the air chambers were free and have no loading force. The measurements of the sensors were close to zero (the non-zero measurements were due to the dead band errors). When the exoskeleton was worn on the subject and the thigh frame was fastened by the fastening strap, the two chambers were both pressured and the values were close to 6 N.

**Figure 14:**
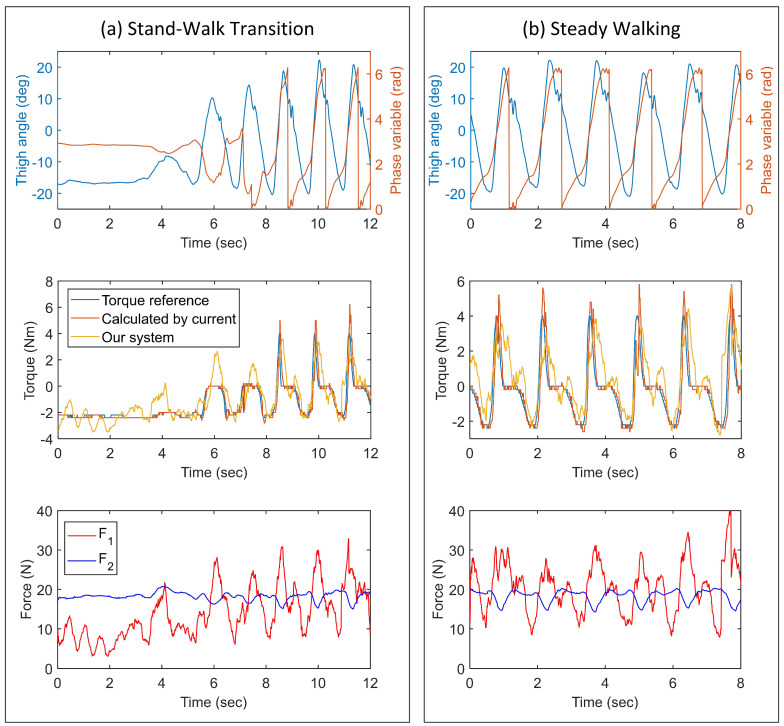
Torque measurements of hip exoskeleton experiments ( $\mu_{1}$ = 0.45, $\mu_{2}$ = 0.65, $\sigma_{1}$ = 15, $\sigma_{2}$ = 10).

When the exoskeleton generated assistive torques, the loading forces of the air chambers were changed under the action of the thigh frame of the exoskeleton. The gait motion and measurements of the forces and torques are shown in Figure 14. Figure 14a shows a stand-to-walk transition process, and Figure 14b shows the steady-walking process. The data in Figure 14 was tested form Sub. 1 in Table 2 and the data of other subjects were tested similarly.

**Figure 15:**
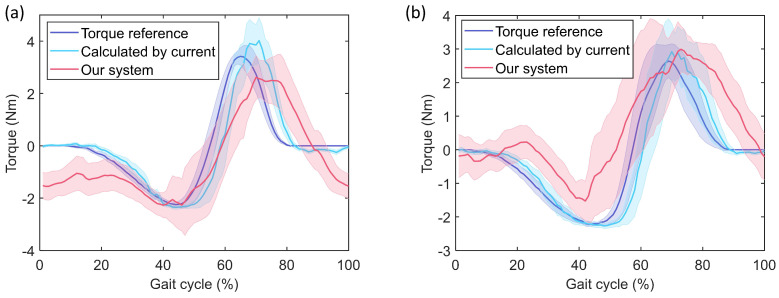
Results of hip exoskeleton test experiments. Experimental settings: (**a**) $\mu_{1}$ = 0.45, $\mu_{2}$ = 0.65, $\sigma_{1}$ = 12, $\sigma_{2}$ = 6; (**b**) $\mu_{1}$ = 0.45, $\mu_{2}$ = 0.65, $\sigma_{1}$ = 15, $\sigma_{2}$ = 10. The shadowed areas in the figure represent ± one standard deviations.

The statistical results (steady-walking) of different exoskeleton assistance torques on the treadmill are reported in Figure 15. It is worth noting that the torque measurement results obtained by the soft force sensor reflect the interaction force between the exoskeleton and the human body. The assistive torque value of the motor was calculated by the current. As shown in Figure 15, under different assistive torque profiles, the torques calculated by motor current is similar to the command torques. The timing and amplitude of assistive torque greatly affect the performance of exoskeleton. However, the interaction force between exoskeleton and human thigh cannot be monitored through the current-based torque calculation method. Based on the developed soft sensor system, the effect of assistance can be reflected through the direct measured interactive force. The results above exactly confirm the necessity of online direct measurement of the human-exoskeleton interaction force.

## 4. Discussion

In this paper, a soft force sensor was designed and integrated into a hip exoskeleton to measure human-exoskeleton interaction force directly. The hip exoskeleton for gait assistance generally can generate a maximum torque within 10 Nm. For example, the maximum torque was set to 6 Nm in [11], and that was set close to 8 Nm in [28]. Thus, the interactive force range is estimated to be 0 to 50 Nm according to the size of the human body. The sensor designed in this paper is able to measure the loading force with a range between 5 N to 80 N, which covers the force requirements of gait assistance hip exoskeletons. The comparisons between the developed sensor system in this paper and relevant studies are shown in Table 3.

In the experiments, we tested different torque profiles of the exoskeleton. The timing of the assistance output torque was different in each mode. These differences were reflected in the results based on the developed soft sensor system. However, the torque monitoring method based on current can not directly measure the change of human-robot interaction force. This provided us with a better understanding of the importance of directly measuring the interaction force between the exoskeleton and the human body since the wrong timing may hinder the user’s movements. From this point of view, the control performance of partial-assist exoskeletons may be improved by introducing the human-exoskeleton interaction force measured directly. Interaction force/torque measurement is suggested to be important to wearable robots [1,29]. As a first attempt, our previous work [25] demonstrated the feasibility of introducing human-exoskeleton interaction into exoskeleton control. However, this depends on the sensing system of direct measurement. The work of this paper provides the support for achieving this goal.

Furthermore, compared with traditional rigid force/torque sensors used in exoskeletons [19,20,21], the sensor designed in this paper is light-weight and low-cost, which can improve its usability in wearable robots. Since the torque sensors with light weight and high performance are usually expensive, thus increases the cost of the exoskeleton system. The cost of the sensor system in this work was beyond 40 dollars. Due to the good adaptability to the human body, soft sensors may be widely used in more and more areas of human-robot interaction. Chambers with different materials and thicknesses were tested, and the test results can provide some guidance for similar designs of soft wearable sensors in other applications.

Last but not least, the developed sensor system has potential uses in security monitoring and control of partial-assist exoskeletons based on the experimental results. For example, the interaction force can be directly monitored in real-time for exoskeleton safety control in an emergency situation. Since the measurement accuracy in wearable sensors is less than the requirements of precision measurement in industry, manufacturing these non precision sensors is also competitive in cost.

There are several limitations in this work, for example, the developed sensor system was tested with eight healthy subjects in the experiments. The adaptability of different individuals remains to be evaluated in future work. Experiments with a larger group of people will be helpful to evaluate the performance and to improve the design. Besides, multi-mode locomotion needs to be solved in the real environment. Testing the system developed in this paper in more scenarios is also one of the future works.

## 5. Conclusions

This paper presents a soft pneumatic sensor system for a hip assistance exoskeleton for human-robot interaction force and torque detection. Real-time delivered force can be monitored through a set of air chambers integrated with the exoskeleton thigh frame. Furthermore, the assistive torque can be obtained via calculation based on the torque measurements. The usability of the developed sensor system was demonstrated using a hip joint exoskeleton prototype. Future work will use the sensor system for online monitoring and optimization of the control performance of hip exoskeletons.

## Figures and Tables

**Table 1:** Performance of the developed soft sensor system for force measuring.

Performance Index	Value
Effective measuring range	80 N
Sensitivity	0.019 V/N
Dynamic error (calibrated)	10.3 ± 6.58%
Time delay	≤0.4 s
Weight	15 g

**Table 2:** Subjects’ Information.

Subject	Gender	Age (year)	Height (cm)	Weight (kg)
Sub.1	M	30	162	64
Sub.2	M	25	178	83
Sub.3	F	21	165	52
Sub.4	F	20	158	58
Sub.5	F	25	155	51
Sub.6	M	24	185	87
Sub.7	M	24	178	90
Sub.8	M	26	174	77
Mean ± Std	–	24.4 ± 3.1	169.4 ± 10.8	70.3 ± 15.9

**Table 3:** Comparisons with relevant studies.

Sensor	Applications	Force Range (N)	Subjects
Pneumatic sensor [22]	Human-robot interaction	12.5	—
FSR-based sensor [18]	Hip exoskeleton	25	1
Three-Axis force sensor [23]	Human-robot interaction	13($F_{z}$)	—
Our work	Hip exoskeleton	80	8
